# Death from Opening an Abscess

**Published:** 1881-07

**Authors:** 


Death from Opening an Abscess.—Another illustration of the peculiar dangers in which medical men are exposed in the practice of their profession is afforded by the lamented death of Dr. Ferdinand Jencken, of Kingstown. It appears that on January 1st Dr. Jencken opened an abscess in the arm of a poor woman, having on his thumb at the time of operating a cut or abrasion so slight as to have been entirely overlooked. The hand and arm, however, soon became violently inflamed, symptoms of pyemia rapidly supervened, and death closed the scene. —*London Lancet.*

